# Reconfiguration of clinical training delivery in a family planning and post-abortion care quality assurance programme in Sierra Leone, 2018–2022: a retrospective programme evaluation

**DOI:** 10.1080/16549716.2026.2761762

**Published:** 2026-11-12

**Authors:** Felix Ikenna Onunkwor, Olaiya Paul Abiodun, Rose Aguolu

**Affiliations:** a Medical Development, Marie Stopes International, Freetown, Sierra Leone; b Department of Public Health, Texila American University, Georgetown, Guyana; c Public Health, National Open University of Nigeria, Abuja, Nigeria; d Research, Monitoring and Evaluation, National Agency for the Control of AIDS (NACA), Abuja, Nigeria

**Keywords:** Implementation research, health workforce development, competency maintenance, supportive supervision, service integration

## Abstract

**Background:**

Health programmes often use both centralised courses and embedded clinical training, but longitudinal descriptions of how training arrangements change – and how competency safeguards are maintained – remain limited.

**Objective:**

To characterise training-delivery configurations and their implementation context across centre, outreach and public-sector strengthening (PSS) channels in Sierra Leone from January 2018 to June 2022.

**Methods:**

We undertook a retrospective descriptive programme evaluation of the complete 472-event training register (380 clinical training, seven competency-assessed training-of-trainers and 85 non-competency activities), with event-level stratification by period and service-delivery channel. We interpreted register patterns alongside programme supervision reports, PSS strategy and quality-assurance documents. We did not conduct a controlled effectiveness evaluation.

**Results:**

The register contained 286 events in 2018, 52 in 2019, 39 in 2020, 76 in 2021 and 19 in January–June 2022; 209 occurred in outreach, 172 in centres and 91 in PSS. Across 3,817 participant-episodes, 2,571 were recorded in 387 competency-assessed activities; 2,559 had L1–L3 outcomes and 2,013/2,559 (78.7%) attained Level 1. The 2019 workforce-expansion period featured longer residential courses (median 10 days), whereas other periods featured short, service-linked instruction and refreshers. An external 2020 PSS assessment documented quality safeguards and remaining gaps; no pre-implementation QTA baseline was available.

**Conclusions:**

Training configurations evolved with programme expansion, changing staffing needs and pandemic adaptation rather than a simple linear classroom-to-workplace shift. Competency-based restrictions, supervised practice and a developing government-linked trainer network formed the clinical governance context. Descriptive programme records and external assessment do not isolate the causal effects of training modality.

## Paper Context

- **Main findings**: The programme recorded changes in training duration, cohort structure, instructional purpose and delivery format across 472 events from 2018 to June 2022.
- **Added knowledge**: A reconciled 472-event register distinguishes 380 clinical sessions, seven competency-assessed training-of-trainers events and 85 other activities, with clear competency denominators and contextual evidence on decentralised supervision, service readiness and health system strengthening transition requirements.
- **Global health impact for policy and action**: Training reform should be designed jointly with competency assessment, supervised practice, equipment readiness and government quality-assurance capacity; comparative effectiveness and economic gains remain to be evaluated.

## Background

In-service clinical training is among the most widely used – and most expensive – quality improvement strategies in low- and middle-income country (LMIC) health systems [1–4]. Evidence syntheses consistently find that the design of training delivery matters as much as its content: training that is repetitive, practice-based, and delivered close to the work setting outperforms single-episode centralised instruction [5,6]. Low-dose, high-frequency approaches and structured on-the-job training (OJT) have demonstrated superior skill retention relative to conventional residential workshops [7], and demonstration-based instructional techniques such as the four-step approach provide a validated structure for procedural skills teaching [8]. Yet empirical documentation of how real programmes actually configure and reconfigure training delivery over time – the durations, venues, cohort structures, practice models, and trainer configurations that constitute a programme’s training delivery architecture – remains scarce, particularly for family planning (FP) and post-abortion care (PAC) services in sub-Saharan Africa [9–11].

Understanding delivery configuration matters for planning: residential courses and short workplace-based visits require different combinations of training time, travel and service arrangements. Training programmes may need to adapt their arrangements to workforce needs, service context and disruptions such as COVID-19 [5,12,13].

The Marie Stopes International Sierra Leone (MSSL) programme delivered family planning and post-abortion care (FP/PAC) through centres, mobile outreach and public-sector strengthening (PSS). Training was embedded in competency assessment, workplace mentorship, supportive supervision and service-readiness support. A separately published evaluation of the 2019–2020 PSS capacity-building package reported knowledge, competency, quality-of-care and service-volume trends [14]. Those programme-associated outcomes were not a controlled estimate of training effectiveness, and the present study asks a different question: how training itself was organised and reconfigured across all three delivery channels over 2018–June 2022.

We addressed three descriptive research questions: (1) How did recorded training type, duration, cohort size and channel distribution change during 2018–June 2022? (2) How did programme expansion and pandemic-period delivery adaptations affect training organisation? (3) What training-to-competency, service-readiness and clinical governance mechanisms were documented alongside these changes? We do not estimate the independent effects of training modality on clinical quality, service output or cost.

## Methods

### Study design

We conducted a retrospective descriptive programme evaluation using the complete programme training event register for January 2018–June 2022 (472 recorded events). Events were summarised by year, type, delivery channel, duration, cohort size and recorded competency outcomes. The documentary component comprised a structured, deductive review of 18 quarterly supportive supervision reports for the same period, together with other routine programme summaries. The documents served to contextualise and corroborate register-based patterns rather than to establish causal effects. The three documentary domains were training-delivery models, instructional methods and capacity-building strategies. January–June 2022 represents a partial year. The event register contained no training activities dated April–June 2018 (Q2); routine continuous supportive supervision during that quarter was retained as programme context, not recoded as training. No comparative intervention group or prospective recruitment was included. The five temporal configurations are interpretive descriptors, not prespecified intervention arms (Table 1).

**Table 1.** Interpretive descriptions of annual training delivery configurations.

Era	Period	Dominant format	Duration/cohort	Venue & practice model	Trainer configuration
Embedded individualised	2018	Topic-specific sessions at the provider’s own facility	1–3 days; 1–4 providers	Service delivery point; supervised practice on live clients	Single trainer per event
Centralised workforce expansion	2019	10-day residential classroom courses	10 days; 9–33 participants	Central venue; models plus organised client practice	4–11 trainers per course
Adaptive pivot	2020	Refresher sessions; trainer endorsement; district OJT wave	2–3 days; variable	Mixed; December wave across four districts with public-sector co-facilitation	6–12 trainers incl. district teams
Remote-capable precision	2021	Refresher OJT; remote refreshers; competency verification	1–2 days; 1–4 providers	Service point or remote; smartphone-based support	Localised trainers leading; founders supervising
Localised maintenance	Jan–Jun 2022	1-day targeted refreshers and trainer-led sessions	1 day; 1–4 (clinical)	Service point; verification-oriented	Second-generation leads under light-touch supervision

OJT, on-the-job training.

### Setting

During the observation period, MSSL supported static centres, mobile outreach teams and the PSS channel, working with the Ministry of Health and Sanitation (MoHS) to build competency for FP/PAC provision in government facilities [15–17]. PSS commenced in October 2018 and supported approximately 90 community health centres across 14 districts in the programme context described in the 2020 external assessment. Initial implementation combined formal clinical training with equipment, consumables, supervised practice, mentorship, competency assessment [18] and quality audits. The organisational arrangement included national and district government trainers and MSSL clinical personnel; personnel turnover meant that contemporaneous strategy headcounts were not additional, disjoint populations from the continuously updated trainer database. Programme personnel counts were therefore taken from the register/database where reported, while the dated strategy presentation was used to interpret the structure of decentralisation.

### Data sources

The primary data source was the routinely maintained training-event register. It recorded event type, location, delivery channel, topic, date, duration, participants, trainers, Level 1–3 outcomes and client-service contacts. The register contained 472 entries: 380 clinical training events, seven ToT events and 85 non-competency workshops. All seven ToT events contained recorded Level 1–3 competency outcomes; consequently, clinical training and ToT jointly comprised 387 competency-assessed activities. Summed attendance describes participant-episodes, not unique staff. Three parallel pairs of topic-specific assessments were retained because their distinct topics, trainers and outcomes represented separate recorded activities involving potentially repeated cohorts; counts must not be interpreted as unique persons or distinct calendar-day sessions. Although these checks strengthen internal consistency, independent verification of all events against external schedules was not available. Other quality technical assessment, incident and service datasets were not treated as linked outcomes of this evaluation.

The contextual evidence comprised a programme extraction matrix identifying 18 quarterly supportive-supervision reports for January 2018–June 2022, a January 2021 public-sector partnership strategy presentation, internal Health System Strengthening (HSS) transition guidance, and the March–April 2020 PSS external Quality Technical Assessment (QTA) narrative and facility-score summary. The first author used a structured deductive documentary review of three predefined domains – training-delivery models, instructional methods and capacity-building strategies – to relate implementation documents to the event register. No formal line-by-line coding, independent dual coding or intercoder reliability testing was undertaken. The quarterly extraction matrix was reconciled with the event register; in particular, Q2 2018 was described as routine continuous supportive supervision without documented training events. Documentary findings were treated as contextual evidence, not independent causal tests. The published 2019–2020 PSS operations research evaluation [14] was considered as contextual prior evidence, not pooled into the present training-event analysis.

### Variables and analysis

Reporting was informed by relevant STROBE items for the descriptive observational component.

We tabulated training events, the mutually exclusive categories clinical training/ToT/non-competency workshops, service-delivery channel, year, event duration, participant-episodes and recorded Level 1–3 competency outcomes. Each register row was the unit for event counts; multiple topic-specific events on the same day could include the same cohort and were not interpreted as unique individual providers or distinct daily sessions. The three pairs of parallel topic-specific records had different topics, trainers and competency outcomes and were retained. Annual summaries are descriptive, without adjustment for cohort composition; 2022 represents January–June only. Among 387 competency-assessed activities, Level 1 attainment was the number of Level 1 participant-episodes divided by the total of documented L1, L2 and L3 outcomes. The 12 unclassified participant-episodes were excluded, not treated as failures or retrospectively assumed to be L3. L3 was a service-specific documented assessment level associated with restricted independent practice, whereas a missing assessment could arise during the training-to-assessment pathway; individual reasons were not recorded. Client contacts in the register were not assumed all to be directly observed.

For the February 2021 remote refresher event, live smartphone-enabled audiovisual instruction and feedback from central trainers were combined with physically present clinical oversight by trained, quality-assured regional FP managers. Managers operated within their usual regions and relied on existing programme petty-cash allocations rather than new travel, accommodation or DSA budgets. This is a programme cash-expenditure description, not a full costing study. Structured client feedback was returned by 495 of approximately 1,500 attendees (33%); neither non-respondent characteristics nor an encounter-level indicator of audiovisual versus in-person observation was available. Client-feedback indicators were analysed descriptively, without claims of representativeness or modality-specific clinical outcomes. Structured client feedback was collected using Marie Stopes International’s standardised global Client Feedback Form, an eight-item paper-based self-completion instrument used routinely across MSI programme settings. The form covers service type and seven experience dimensions: respectful treatment, clarity of communication, ease of asking questions, privacy, waiting time, waiting-time explanation, and willingness to recommend the programme, with an optional item on pain management. Items on experience quality (Q1–Q4 and Q7) used a 4-point ordinal scale (Not at all/A bit/Mostly/Definitely); waiting time (Q5) used four ordered categories (less than 15 minutes; 15–30 minutes; 30–45 minutes; more than 45 minutes); waiting-time explanation (Q6) was binary (Yes/No); and pain management (Q8) offered three response options (Yes/No/I did not feel any pain). The instrument is a programmatic quality-monitoring tool and has not undergone formal psychometric validation. Positive responses were defined as Mostly or Definitely for Q1–Q4 and Q7, less than 30 minutes for Q5, and Yes for Q6. Results are interpreted strictly as descriptive respondent-reported feedback, subject to selection, nonresponse and social-desirability biases.

### Ethical considerations

Ethical and scientific approval was granted by the Sierra Leone Ethics and Scientific Review Committee (SLESRC), Directorate of Training and Research, Ministry of Health and Sanitation, Government of Sierra Leone, for the study entitled ‘Piloting Remote Approach to Clinical Assessment’. Approval was initially granted on 7 July 2020 and subsequently extended through 20 July 2023 (SLESRC Reference No. 008/09/2022). The approved research concerned remote clinical assessment within Marie Stopes Sierra Leone’s integrated clinical quality-assurance system. Quality Technical Assessment and Clinical Quality Internal Audit drew on related operational sources, including clinical training and competency records, supportive supervision reports, service-delivery information, client feedback and clinical-governance documentation, to inform assessment, supervision and corrective action. The present study retrospectively analysed these pre-existing routine programme records. The continuous training register was generated contemporaneously through normal programme activities, including historical baseline periods, rather than reconstructed specifically for research. The analytical records were accessed for research purposes on 20 December 2022 during the approved extension period. Marie Stopes Sierra Leone separately granted institutional permission and data-use authorisation on 30 May 2021 for use of relevant de-identified quality-assurance, service-delivery, staff competency and training, and implementation records for doctoral research and related scholarly publication, subject to confidentiality restrictions. Routine client feedback was collected in the course of service delivery and analysed retrospectively in aggregate. No individuals were recruited or contacted specifically for this analysis, and no biological specimens were collected. Although source registers contained trainer names, these identifiers were omitted from de-identified analytical sharing copies and are not disclosed in reported findings. The study adhered to applicable ethical principles, including the Declaration of Helsinki.

## Results

### Overview of the register

The register documented 472 training activities: 380 competency-based clinical training events (80.5%), seven competency-assessed training-of-trainers events (1.5%) and 85 non-competency activities or workshops (18.0%). The 387 competency-assessed activities contributed 2,571 participant-episodes; 2,559 had end-of-training Level 1–3 outcomes (99.5%), including 2,013 Level 1 outcomes (78.7% of classified episodes). Twelve participant-episodes in 10 clinical training records (eight in 2021 and four in January–June 2022) lacked a recorded level. No ToT event accounted for these unclassified outcomes. The whole register comprised 3,817 participant-episodes and 30,790 recorded client-service encounters. By delivery channel, 209 events were in outreach, 172 in static centres and 91 in public-sector strengthening. Recorded client encounters do not necessarily represent directly observed procedures. Table 2 reports year-specific descriptive measures; 2022 covers January–June only.

**Table 2.** Training delivery architecture by year for the MSSL Sierra Leone FP/PAC programme (January 2018–June 2022; *N* = 472 events).

Indicator	2018	2019	2020	2021	Jan–Jun 2022
Training events (n)	286	52	39	76	19
Participant-episodes (n)	957	912	1,068	643	237
Median duration, days (IQR)	1 (1–2)	10 (3–10)	2 (2–3)	2 (1–2)	1 (1–1)
Events ≤ 2 days (%)	88	13	74	76	79
Events ≥ 10 days (%)	0	62	0	3	0
Median cohort size	2	14	24	4	16 a
Median trainers per event	1	4	6	1	4
Refresher events (%)	0	0	51	82	74
Remote events (n)	0	0	0	5	0
Recorded client-service encounters (n)	10,862	9,025	6,032	4,612	259
Level 1 (n/N, %) b	491/799, 61%	595/737, 81%	561/593, 95%	334/379, 88%	32/51, 63% c

a: January–June 2022 is a partial year; the median cohort size refers to all registered event types, not only competency-assessed clinical training. b: Annual Level 1 n/N figures use the sum of recorded Level 1–3 outcomes among assessed participant-episodes as denominator, excluding unclassified episodes and non-competency workshops; percentages are rounded to the nearest whole number. c: The 2022 Level 1% is based on January–June assessments only; it is not an annualised estimate. Rounded annual percentages are 61%, 81%, 95%, 88% and 63%. IQR, interquartile range.

## Training category and channel stratification

Clinical training (380) and ToT (7) were kept distinct while recognising that both contained end-of-training competency-assessment records; 85 remaining events were non-competency activities. The channel totals – 209 outreach (44.3%), 172 centres (36.4%) and 91 PSS (19.3%) – describe all event types, not exposure to a common intervention. Event purpose, duration and participant mix changed over time, including a concentration of longer workforce-expansion courses in 2019 and shorter targeted refreshers thereafter. Where an event lacked an unambiguous initial/refresher label, such status was not inferred from its title alone. The register contained no documented training events in Q2 2018; this absence of registered training did not imply an interruption of routine supportive supervision.

### The 2019 centralised wave as the architectural anomaly

The 2019 records differed from those of other years: median event duration was 10 days (IQR 3–10), and 62% of events lasted at least 10 days. During public-sector workforce expansion, the register included thirty 10-day residential courses for Ministry of Health providers. In 2018, 286 recorded events were predominantly brief, facility-based and small-cohort.

### The 2020 adaptive pivot

Three distinctive activities were recorded in 2020: refresher events comprised 51% of events; one seven-day trainer-endorsement event assessed 32 trainers at Level 2; and sixteen two-day workplace events in December involved 628 participant-episodes and 5,069 registered client encounters across four districts, with participation by district teams. Median event duration was 2 days. The register reported 95% Level 1 attainment among assessed participants in competency-based events.

### 2021–2022: remote capability, precision, and localisation

In 2021, refresher events comprised 82% of registered events, and five events were recorded as remote (123 participant-episodes). One February 2021 remotely supported counselling refresher was linked to approximately 1,500 service-point client contacts, 21 participant-episodes and five trainers. Central trainers used live smartphone-enabled audiovisual communication for instruction, provider demonstration and feedback. Trained and quality-assured regional Family Planning managers were physically present within their regions to provide on-site clinical oversight, risk mitigation and capacity to intervene as necessary during practical activities. Remote trainer observation should not be equated with direct physical observation, nor does the register identify which client contacts were observed in either way. Regional managers used already established programme resources, including routine petty-cash allocations that would have existed independently of the remote event; their participation did not require additional per diem, accommodation or DSA allocations. This describes the incremental cash-budget arrangement, not a full costing or a measured net cost saving. Forty-three competency-based refresher events involved four or fewer participants over four or fewer days. The annual training-delivery configurations across all five periods are summarised in Table 1.

### Documentary findings from supportive supervision and programme records

Documentary analysis identified an implementation pathway linking initial teaching, supervised clinical practice, service-specific assessment and continuing mentoring. The HSS transition guidance specified that government providers independently delivering particular FP/PAC services should be assessed as Level 1 competent for those services; transition from direct to indirect support additionally required competency coverage, clinical quality, data quality and commodity-readiness criteria. The 2021 PSS partnership presentation documented national, district and MSSL training and supervision structures and envisaged progressively greater MoHS responsibility. These are written organisational requirements and proposed arrangements – not proof of full compliance or of facility graduation. The programme also accommodated newly recruited, locum and service-specific L3 providers through nearby OJT and reassessment. Historical staffing and programme experience make this a plausible explanation for training attendance without contemporaneous classification, but do not establish the reason for each of the 12 unclassified participant-episodes.

The PSS programme illustrates why training delivery cannot be analysed in isolation from service readiness. According to programme implementation records and experience, participating public-sector sites were selected in part because they lacked providers trained for the relevant FP/PAC services and the essential equipment needed to initiate those services. The implementation package therefore comprised initial provider training followed by equipment, consumables and job aids, with supervised clinical practice, method-specific competency assessment and MSSL/MoHS master-trainer mentorship and subsequent supervision. In such circumstances, a directly comparable pre-intervention QTA of clinical performance for services not yet operational would not have been feasible; this is distinct from a pre-intervention assessment of facility readiness. The 2020 external QTA report documented supportive-supervision registers, competency records, provider observation and quality-audit action plans, as well as provider transfers, equipment shortfalls and difficulties maintaining aseptic technique when a single provider was onsite. Thus the evidence concerns an integrated capacity-building and service-readiness package, not an isolated effect of classroom or workplace training.

A previously published peer-reviewed evaluation of the PSS programme reported longitudinal programme-level indicators from 2019–2020: mean knowledge-test scores of 28% before and 81% after training; Level 1 competency proportions of 59% in Q3 2019 and 97% in Q4 2020; model-site status of 3% and 33%, respectively, in 2019 and 2020; model-area status of 8% and 22%; and recorded PAC service volume of 442 and 1,950 [14]. These are previously published findings from different source records, time points and denominators, not new analyses of the 472-event register and not measurements of a common cohort of the nine externally assessed facilities. They provide longitudinal programme context, not a substitute for site-matched pre-implementation QTA scores or a controlled estimate of training effects.

Within-period records from the programme’s concurrent Clinical Quality Internal Audit (CQIA) system are available for 29 PSS sites across 10 of 14 programme districts, assessed between September 2021 and June 2022 (the final 21 months of the study period). Assessments were conducted in person by cross-regional assessors paired with MOHS clinical quality assessors, using a standardised multi-domain instrument covering clinical governance, core care, clinical services, clinical record audit, and safety (infection prevention and medical emergency management). De-identified aggregate domain scores from these assessments showed clinical governance (93–96%), core care (92–95%) and clinical services (92–94%) consistently exceeding 90% across both assessment sub-periods, while safety was the lowest-scoring domain (86–87%), corroborating the persistent infection-prevention and emergency-preparedness gaps identified in the 2020 QTA. Of the assessed domains, clinical services is notable in that the CQIA instrument employs the same assessment checklist used during training events to classify individual providers as Level 1, 2, or 3 competent; for this domain specifically, CQIA scores directly reflect performance against the same operational criteria recorded in the training register’s competency classifications, providing methodological coherence between the two data streams. Other domains – clinical governance, core care, clinical record audit, and safety – are assessed at site level only and do not have an equivalent individual competency checklist. The proportion of assessed sites meeting model-site criteria rose from 18% (2 of 11 sites, September–October 2021) to 39% (7 of 18 sites, January–June 2022), consistent with a quality improvement trajectory across the final observation period. These concurrent quality data, provided in de-identified aggregate form (see Supplementary Material 4, S4-12), should be interpreted with the following caveats: assessments covered approximately one-third of the approximately 90 PSS sites; no comparable CQIA records for the PSS channel were available before September 2021 within the programme database; and CQIA is an internal monitoring instrument rather than an independent external assessment.

### External triangulation

An external PSS QTA was conducted from 23 March to 9 April 2020, after the PSS channel began in October 2018 and after initial provider training, supply of essential equipment and consumables, and commencement of supervised service delivery. Eleven sites were selected and nine were assessed; two could not be visited owing to COVID-19 restrictions. Programme management recalls random selection, but the available narrative does not specify the sampling frame or selection procedure. The nine-site summary identified three model sites, high scores in several assessed domains, and persistent infection-prevention and staffing gaps. The initial lack of trained providers and essential service equipment helps explain why comparable pre-intervention clinical-performance QTA scores were unavailable for these newly supported sites: the relevant services had not yet been established on a comparable basis. This does not imply the absence of earlier programme-level monitoring; a published PSS evaluation reported separate 2019–2020 trends [14]. The external QTA provides an independent post-implementation quality snapshot, not a matched facility-level estimate of change, evidence of outcomes across all approximately 90 PSS-supported sites, or proof of a causal training effect.

### Client experience during OJT events

During the February 2021 remotely supported refresher period, 495 of approximately 1,500 clients attending participating service-delivery points completed routine structured feedback forms (approximately 33%). Among respondents, 100% reported understanding the provider; 95% found it easy to ask questions; 90% indicated adequate privacy; 89% reported respectful care; 89% reported satisfaction with pain management; and 85% indicated they would recommend the programme. Twenty-five respondents (5%) reported waiting more than 30 minutes; 75% reported positively regarding waiting-time notification. These outcomes describe respondents rather than all attendees: non‑respondent characteristics and reasons for nonresponse were unavailable, and selection, nonresponse and courtesy biases cannot be excluded. Feedback records did not identify whether a particular encounter was audiovisually observed, directly overseen on site, or not observed for training.

## Discussion

The central finding is a reconfiguration of training delivery, not a demonstrated treatment effect. Across 2018–June 2022, the programme alternated between short workplace-linked instruction, longer centralised courses during the public-sector expansion, locally supported refreshers and selective audiovisual teaching during COVID-19. The event-level evidence therefore qualifies a simple narrative that classroom teaching was wholly replaced by OJT; different formats coexisted and served different staffing and programme purposes.

The pronounced 2019 residential-training pattern coincided with a large PSS capacity-building initiative and should be interpreted as a workforce-expansion activity, not a programme-wide withdrawal from embedded practice. The prior PSS study [14] analysed knowledge acquisition, clinical competency, facility quality and PAC service trends during 2019–2020. By contrast, this analysis covers the wider register across all three channels and examines delivery architecture rather than re-reporting PSS outcome trends. Annual Level 1 proportions in this paper refer to assessed participant-episodes in training events and should not be equated with the earlier paper’s quarterly measures of assessed providers.

The external QTA found favourable assessed domains but identified continuing infection-prevention, documentation and skill-maintenance needs; concurrent CQIA records from 2021–2022 similarly identified safety (infection prevention and medical emergency management) as the lowest-scoring domain (S4-12), providing independent corroboration from internal quality monitoring. This underscores that training must be accompanied by service readiness, commodities and follow-up.

The 2021 remotely supported refresher illustrates a hybrid approach: central trainers used audiovisual instruction while trained, quality-assured regional FP managers remained physically present and able to intervene. Existing regional operating arrangements avoided additional dedicated travel allowances for those managers. Thus the programme experience supports a hypothesis about reduced incremental cash outlays, not a formal finding of cost savings. Client feedback from 495 of approximately 1,500 contacts was descriptive, with substantial nonresponse uncertainty. Comparative effectiveness, safety, staffing and full economic costs require prospectively designed evaluations.

### Strengths and limitations

The study benefits from register-wide coverage of 472 recorded events, clear separation of 380 clinical/7 ToT/85 workshop activities, explicit participant-episode denominators, and triangulation with PSS strategy, clinical governance and external assessment records. Limitations include: retrospective administrative coding and possible duplicate cohort participation; 12/2,571 competency-event participant-episodes lacking a documented L1–L3 outcome; no reliable individual longitudinal linkage or consistent baseline competency; uncalibrated trainer-scored competency proportions; changing case mix, event purpose and service channel; an incomplete 2022 observation year; no comparator or adjusted causal identification; incomplete service-specific observation for external QTA; and non-response in client feedback. The external PSS QTA assessed only nine of 11 selected facilities and lacked comparable pre-implementation site-specific clinical-performance QTA scores; selection was described as random from programme recollection, but documentation did not establish the procedure. Policy guidance expresses intended standards, not audited adherence, and the documentary interpretation was undertaken by the first author without independent coding. The paper also shares implementation context with an earlier peer-reviewed PSS evaluation [14], although its research question, period and training-register analysis differ.

## Conclusion

Across 472 registered activities in Sierra Leone from January 2018 through June 2022, clinical training delivery evolved in response to public-sector expansion, local staffing needs and the COVID-19 period. Long residential courses and brief workplace-based instruction were complementary configurations rather than sequentially exclusive models. Documented competency requirements, supervised practice, distributed trainer structures and service-readiness support explain how decentralisation was intended to operate within clinical quality governance. The study offers a transferable implementation description [19] and explicit analytical definitions, but cannot demonstrate that any single delivery mode caused improvements in clinical quality, safety, efficiency or health-system sustainability [20,21].

## Supplementary Material

GHA_268442593_S2_Supplementary_Evidence clean.docx

GHA_268442593_S3_PSS_QTA_Supplement_clean.docx

GHA_268442593_S4_Clinical_Governance_Evidence_clean.docx

GHA_268442593_STROBE_Checklist.docx

## Data Availability

The original training event register contains staff-identifying and confidential programme information and is not publicly available in identifiable form. A de-identified event-level analytical dataset of 472 recorded training events and aggregate supporting tables has been prepared in accordance with the institutional permission and data-use authorisation granted by Marie Stopes Sierra Leone on 30 May 2021. Access to de-identified analytical records may be requested from the corresponding author on reasonable request and is subject to the confidentiality and data-use conditions of that authorisation, including appropriate safeguards against indirect identification. The underlying supportive supervision reports and individual client feedback records are confidential programme documents and are not publicly available. No unrestricted public redistribution of original programme records is authorised by this statement.
