# Supplementary material for: Reconfiguration of clinical training delivery in a family planning and post-abortion care quality assurance programme in Sierra Leone, 2018–2022: a retrospective programme evaluation
Source: Glob Health Action. 2026 Nov 12;19(1):2761762. doi: 10.1080/16549716.2026.2761762 (PMC13761034; doi:10.1080/16549716.2026.2761762)
Supplement: Supplementary file 1 [file ZGHA_A_2761762_SM0832.docx]

STROBE checklist — descriptive observational programme evaluation

Manuscript: Reconfiguration of clinical training delivery in a family planning and post-abortion care quality assurance programme in Sierra Leone, 2018–2022. Manuscript ID: GHA 268442593.

Locations refer to manuscript section headings; page numbers are checked against the accompanying manuscript PDF rendering. “Not applicable” is used where the standard STROBE item assumes a cohort, case-control, or comparative effect analysis not undertaken here.

| STROBE item | Item no. / reporting recommendation | Reported in manuscript | Status / clarification |
| --- | --- | --- | --- |
| Title/abstract | 1(a). Indicate study design in title or abstract | Title; Abstract | Retrospective programme evaluation stated in title and abstract |
| Title/abstract | 1(b). Provide informative, balanced summary | Abstract (p. 1) | Design, register counts, outcomes, limitations and conclusion |
| Background | 2. Explain scientific background and rationale | Background (p. 1, 2) | Training architecture, competency safeguards, implementation gap |
| Objectives | 3. State specific objectives and prespecified hypotheses | Background; Abstract (p. 1, 2) | Three descriptive research questions; no causal hypothesis tested |
| Study design | 4. Present key elements of study design early | Methods—Study design (p. 2) | Retrospective descriptive register evaluation plus deductive documentary review |
| Setting | 5. Describe setting, location, periods and relevant dates | Methods—Setting; Study design (p. 2, 3, 13) | Sierra Leone; three channels; Jan 2018–Jun 2022; partial year explicit |
| Participants | 6(a). Give eligibility criteria, sources and methods of selection | Methods—Study design; Data sources (p. 2) | Census of registered programme training events rather than sampled individuals |
| Participants | 6(b). For matched studies give matching criteria; for cohorts follow-up methods | Methods—Study design; Data sources (p. 2) | Not applicable: no matched cohort, follow-up sample or case-control design |
| Variables | 7. Define outcomes, exposures, predictors and confounders | Methods—Variables and analysis; Data sources (p. 4) | Training categories, channels, event duration, participant-episodes, L1–L3 |
| Data sources | 8. Describe sources and measurement methods; comparability where relevant | Methods—Data sources; Variables and analysis (p. 3) | Register, 18 quarterly reports, QTA narrative and programme documents; distinct measures |
| Bias | 9. Describe efforts to address potential sources of bias | Methods—Variables and analysis; Strengths and limitations (p. 4) | No unique-person linkage, compositional confounding, partial year, client feedback selection |
| Study size | 10. Explain how study size was arrived at | Methods—Study design; Results—Overview (p. 5) | Complete 472-event register; no sample-size calculation for census |
| Quantitative variables | 11. Explain handling of quantitative variables and groupings | Methods—Variables and analysis; Table 1 (p. 4) | Annual and channel grouping, Level 1 numerator/denominator, missing classifications |
| Statistical methods | 12(a). Describe statistical methods including control of confounding | Methods—Variables and analysis (p. 4) | Descriptive aggregation only; no adjusted inference or confounder control |
| Statistical methods | 12(b). Describe methods to examine subgroups and interactions | Results—Training category and channel stratification (p. 5) | Descriptive year/category/channel stratification; no interaction models |
| Statistical methods | 12(c). Explain how missing data were addressed | Methods—Variables and analysis; Results—Overview (p. 5) | 12/2571 unclassified outcomes excluded from 2559 classified denominator |
| Statistical methods | 12(d). Explain handling of loss to follow-up | Methods—Study design (p. 2) | Not applicable: no individual follow-up cohort |
| Statistical methods | 12(e). Describe sensitivity analyses | Methods—Variables and analysis (p. 4) | Not conducted; observational descriptive design; no inferential effect estimates |
| Participants | 13(a). Report numbers at each stage | Results—Overview; Table 1 (p. 5) | 472 events; 387 competency-assessed; 2571 episodes; 2559 classified |
| Participants | 13(b). Give reasons for nonparticipation at each stage | Results—Overview; Strengths and limitations (p. 5) | All register events included; 12 episode classifications unavailable, reasons not documented |
| Participants | 13(c). Consider flow diagram | Results—Overview (p. 5) | No diagram; event/episode accounting reported in text and Table 1 |
| Descriptive data | 14(a). Give participant characteristics and exposure information | Table 1; Results—Training category and channel stratification (p. 5) | Only event-/episode-level features available; unique participant demographics unavailable |
| Descriptive data | 14(b). Indicate number missing for each variable of interest | Results—Overview; Methods—Variables and analysis (p. 4) | 12 missing competency classifications; other variable-specific missingness not fully tabulated |
| Descriptive data | 14(c). For cohorts summarise follow-up time | Methods—Study design (p. 2) | Not applicable: not an individual-level longitudinal cohort |
| Outcome data | 15. Report numbers of outcome events or summary measures | Results—Overview; Table 1 (p. 5) | 2013/2559 Level 1, annual proportions; descriptive training event counts |
| Main results | 16(a). Give unadjusted and adjusted estimates with precision | Results; Table 1 (p. 1, 5) | Unadjusted descriptive proportions; adjusted effect estimates and CIs not calculated |
| Main results | 16(b). Report category boundaries when continuous variables categorised | Table 1; Methods—Variables and analysis (p. 4) | ≤2 days and ≥10 days explicitly labelled; median/IQR reported |
| Main results | 16(c). Consider translation of relative risk to absolute risk | Methods—Study design (p. 2) | Not applicable: no relative risk or intervention-effect estimates |
| Other analyses | 17. Report subgroup, interaction or sensitivity analyses | Results—Training category and channel stratification; Table 2 (p. 5) | Channel/year/category descriptors; no interaction or sensitivity analyses |
| Key results | 18. Summarise key results relative to objectives | Discussion; Conclusion (p. 9) | Reconfiguration not replacement; safeguards part of implementation system |
| Limitations | 19. Discuss limitations, bias directions and imprecision | Strengths and limitations (p. 10) | Retrospective coding, outcome denominator, external QTA sampling, lack of baseline/adjustment |
| Interpretation | 20. Give cautious overall interpretation considering evidence | Discussion; Conclusion (p. 9) | No causal modality claims, cost-effectiveness claims or facility graduation claims |
| Generalisability | 21. Discuss external validity | Discussion; Strengths and limitations (p. 9) | Contextual Sierra Leone programme; no population-wide inference |
| Funding | 22. State funding source and role of funders | Funding information (p. 12) | No specific grant reported |

Reporting qualification: STROBE is a reporting checklist, not a certification of study quality. The descriptive design does not support adjusted modality effects. Full variable-specific missingness beyond the 12 unclassified competency outcomes should be verified before any assertion of complete missing-data reporting.
