# Supplementary material for: Reconfiguration of clinical training delivery in a family planning and post-abortion care quality assurance programme in Sierra Leone, 2018–2022: a retrospective programme evaluation
Source: Glob Health Action. 2026 Nov 12;19(1):2761762. doi: 10.1080/16549716.2026.2761762 (PMC13761034; doi:10.1080/16549716.2026.2761762)
Supplement: Supplementary file 2 [file ZGHA_A_2761762_SM0833.docx]

Supplementary File S4

Integrated programme implementation and clinical governance evidence matrix

Global Health Action | Manuscript 268442593 | Sierra Leone FP/PAC programme, 2018–June 2022

# Purpose and scope

This source-indexed synthesis supports interpretation of the 472-event descriptive training-register study. It combines only de-identified programme-level facts, documented organisational requirements, contemporaneous strategy statements, published contextual evidence and transparent implementation accounts. It is not an independent impact evaluation and does not reproduce confidential source documents.

# Evidence classification

Written organisational requirement = intended standard, not measured compliance. Recorded event evidence = administrative record, not proof of unique persons or observed clinical procedures. External assessment = observed post-implementation quality snapshot, not matched baseline. Author/programme account = implementation description needing documentary confirmation where not directly recorded. Previously published evidence remains analytically separate.

# Cross-reference note Initial PSS service-readiness context (including the initial capacity gap, the March–April 2020 external quality assessment and the previously published 2019–2020 programme-level trends) is documented in Supplement S3. The components below cover clinical governance mechanisms, competency standards, trainer structures and operational implementation detail that are not duplicated in S3. Clinical training governance and implementation mechanisms

## S4-01 | Training-to-practice package

**Evidence class:** Documented programme design and practice

**Source locator:** 2020 PSS QTA narrative; revised manuscript, Setting and Documentary findings

**Supported evidence:** Initial instruction was coupled with equipment, consumables, practical support, mentorship, competency assessment and subsequent clinical audit/supervision.

**Interpretive scope:** Integrated intervention; no component-level effect can be isolated.

**Limitation:** Cannot attribute quality status to OJT or equipment alone.

**Relevance to reviewer response:** Discussion of training reconfiguration

## S4-02 | Independent practice competency restriction

**Evidence class:** Written organisational requirement

**Source locator:** HSS Graduation Pathway Guidance, Direct/Indirect Support clinical sections; revised manuscript, Documentary findings

**Supported evidence:** Government providers delivering specified FP/PAC services independently should meet service-specific Level 1 competency requirements.

**Interpretive scope:** Normative institutional guidance, not empirical compliance assessment.

**Limitation:** Not evidence all providers met Level 1 at all points.

**Relevance to reviewer response:** Ethics/safety and competency safeguard questions

## S4-03 | Remediation and reassessment

**Evidence class:** Documented implementation description

**Source locator:** Revised manuscript, Variables and analysis; Documentary findings

**Supported evidence:** Supervised development and repeat assessment supported providers with gaps; recorded L3 is a service-specific assessment status, not the same as a missing classification.

**Interpretive scope:** Programme pathway; no individual follow-up dataset here.

**Limitation:** Do not infer the reason for 12 unclassified participant-episodes.

**Relevance to reviewer response:** Competency denominator clarification

## S4-04 | Transition to indirect support

**Evidence class:** Written organisational threshold

**Source locator:** HSS Graduation Pathway Guidance, transition criteria

**Supported evidence:** Guidance specified a minimum direct-support period and criteria including >=90% active-provider competency coverage and >=65% model areas, plus other quality/data/commodity checks.

**Interpretive scope:** Proposed criteria, not measured transition success.

**Limitation:** No programme-wide graduation counts or verified adherence available from these source documents.

**Relevance to reviewer response:** Sustainability and governance

## S4-05 | Government trainer and supervision structure

**Evidence class:** Contemporaneous strategy / organisational intent

**Source locator:** Establishing new public sector partnership models (26.01.21), government partnership and trainer-network slides

**Supported evidence:** Strategy described national and district MoH trainers, district health sisters and MSSL trainers, with a planned shift toward government-led supervision.

**Interpretive scope:** Historic snapshot and strategic intent.

**Limitation:** Historic headcounts overlap with evolving workforce; do not add to updated trainers database or assert achieved handover.

**Relevance to reviewer response:** Decentralisation and ownership

## S4-06 | Trainer endorsement wave

**Evidence class:** Recorded training event evidence

**Source locator:** 472-event training register; revised manuscript, The 2020 adaptive pivot

**Supported evidence:** One seven-day endorsement activity in 2020 recorded 32 trainers at Level 2.

**Interpretive scope:** Descriptive register entry.

**Limitation:** Level 2 endorsement does not equate to Level 1 clinical competence; do not infer later individual independence.

**Relevance to reviewer response:** Trainer capacity

## S4-07 | December 2020 district-level delivery

**Evidence class:** Recorded training event evidence

**Source locator:** 472-event register; revised manuscript, The 2020 adaptive pivot

**Supported evidence:** Sixteen two-day workplace events in four districts recorded 628 participant-episodes and 5,069 client-service encounters with district-team involvement.

**Interpretive scope:** Event records, not unique trainees or directly verified observations.

**Limitation:** Recorded contacts are not necessarily observed procedures.

**Relevance to reviewer response:** Decentralised scale

## S4-08 | Remote-plus-onsite clinical oversight

**Evidence class:** Implementation description; register event count

**Source locator:** 472-event register; revised manuscript, Variables and analysis and 2021–2022 results

**Supported evidence:** Five 2021 remote-designated events; one February refresher used smartphone audiovisual inputs from central trainers alongside physically present quality-assured regional FP managers.

**Interpretive scope:** Hybrid oversight model, not remote-only surgery supervision.

**Limitation:** No direct modality equivalence, comparative safety effect or contact-level supervision indicator.

**Relevance to reviewer response:** Remote practice and safeguards

## S4-09 | Regional cash budget

**Evidence class:** Author/programme operating account

**Source locator:** Revised manuscript, Variables and analysis, 2021–2022 results, Discussion

**Supported evidence:** Regional FP managers used ordinary regional operating arrangements rather than dedicated new accommodation, travel or DSA allocations for the February event.

**Interpretive scope:** Incremental cash-arrangement statement.

**Limitation:** Not a formal economic costing or net-savings estimate.

**Relevance to reviewer response:** Efficiency reviewer questions

## S4-10 | Client experience and feedback limitations

**Evidence class:** Routine aggregate service feedback

**Source locator:** Revised manuscript, Client experience during OJT events

**Supported evidence:** 495 responses among approximately 1,500 attendees (33%); descriptive indicators, without encounter-level distinction between remote and direct observation.

**Interpretive scope:** Respondent-only descriptive data.

**Limitation:** Selection, courtesy and nonresponse bias; avoid claims of preserved safety or equivalence.

**Relevance to reviewer response:** Acceptability

## S4-11 | Quarterly supervision cross-check

**Evidence class:** Derived documentary extraction

**Source locator:** MS15_SupervisionReport_ExtractionMatrix_Q2_2018_Corrected.xlsx; GHA_SupervisionAnalysis_Q2_2018_Reconciled.xlsx

**Supported evidence:** Q2 2018 records routine supportive supervision but the 472-event register contains no dated training events for that quarter.

**Interpretive scope:** Cross-source reconciliation from available extraction.

**Limitation:** Original set of 18 supervision reports not independently re-audited in full.

**Relevance to reviewer response:** Longitudinal documentary integrity

# Specific safeguards against overinterpretation

- The 2019–2020 published PSS trends and nine-site external QTA are separate datasets with different units and denominators; no matched pre/post facility QTA is implied.
- Absence of trained personnel and essential equipment provides important service-readiness context. A conventional clinical-performance baseline was not directly comparable before service establishment; this does not mean that all other possible readiness baselines were impossible.
- Level 1 policy thresholds and HSS graduation criteria are standards and plans, not verified achievement.
- The recorded 30,790 client-service encounters over the event register are not synonymous with clinically observed procedures or unique persons.
- The documentary evidence matrix is an interpretive synthesis; it is not a substitute for an independently revalidated 18-report corpus.

# Disclosure and source-verification status

No individual staff, provider, client or site is named in this supplementary matrix. The document avoids precise facility identifiers. This does not constitute a formal institutional approval to publish. The underlying HSS guidance, government-partnership slides and original source records are internal documents and are not appended here. Institutional review of disclosure suitability is required before upload. Individual statements should be cross-checked against the cited originals by the authors before final submission.

# Suggested citation in manuscript/rebuttal

Supplementary File S4 provides a structured, de-identified evidence map of programme delivery mechanisms, service-readiness support, competency safeguards and decentralised supervision. It distinguishes normative programme guidance from observed events and external assessment evidence.

## S4-12 | Internal clinical quality assessment (CQIA) results, PSS channel, within study period (September 2021–June 2022)

***Evidence category:*** *Recorded event evidence (concurrent internal programme monitoring system)*

A concurrent clinical quality-assessment system — the Clinical Quality Internal Audit (CQIA) — operated as a standing quality monitoring mechanism throughout the PSS channel during the programme. CQIA assessments were conducted in person using a standardised multi-domain instrument covering: clinical governance, core care (counselling, privacy, and comprehensive contraceptive counselling), clinical services, clinical record audit, safety (infection prevention and medical emergency management), and an overall composite final score. Of note, the clinical services domain of the CQIA instrument employs the same assessment checklist as the individual provider competency assessment administered during training events to classify providers as Level 1, 2, or 3 competent; CQIA service-domain scores therefore directly reflect performance against the same operational criteria recorded in the training register, providing methodological coherence between the two data streams for this domain. Other CQIA domains — clinical governance, core care, clinical record audit, and safety — are assessed at site or facility level and do not have an equivalent individual competency checklist, providing complementary rather than overlapping evidence. Performance meeting pre-defined thresholds across a specified proportion of assessed areas qualified a site as a model site, consistent with the HSS/MSSL model-site framework described in S4-05.

To preserve methodological independence, assessors were drawn from regions other than those being assessed. MOHS clinical quality assessors were actively paired with MSSL programme assessors, providing government co-ownership of the monitoring process and reflecting the collaborative quality-assurance governance described in S4-07 and S4-09.

Within-period CQIA records (January 2018–June 2022) comprised 29 assessments across 29 unique sites, covering 10 of 14 programme districts (Bombali, Bo, Bonthe, Kailahun, Kambia, Kenema, Kono, Pujehun, Western Area Rural, and Western Area Urban). Assessments fell in two sub-periods: September–October 2021 (11 sites; September n=8, October n=3) and January–June 2022 (18 sites; January n=5, February n=10, June n=3). No CQIA records for the PSS channel predating September 2021 were available within the programme's integrated quality database; assessments from July–December 2022 fall outside the study period. Site names and unique identifiers have been suppressed; aggregate domain scores by sub-period are presented in Table S4.1.

***Table S4.1. Aggregate de-identified CQIA domain scores, PSS channel, within study period***

| **Domain** | **Sep–Oct 2021 (n = 11 sites)** | **Jan–Jun 2022 (n = 18 sites)** |
| --- | --- | --- |
| Clinical governance | 96% | 93% |
| Core care (counselling, privacy, CCC) | 92% | 95% |
| Clinical services | 92% | 94% |
| Clinical record audit | 96% (n=6) | 99% (n=15) |
| Safety (infection prevention and MEM) | 87% | 86% |
| Overall final score | — | 93% (n=13) |
| Model site status | 2/11 (18%) | 7/18 (39%) |

*Note: Values are mean percentages calculated from site-level scores within each sub-period. Clinical record audit and overall final score counts are lower than n-sites because some records lacked entries for these fields. Site names, unique identifiers, and assessor names have been fully suppressed. Assessors were cross-regional (not drawn from the district being assessed) and were paired with MOHS clinical quality assessors. CCC = comprehensive contraceptive counselling; IP = infection prevention; MEM = medical emergency management.*

Across both assessment sub-periods, clinical governance, core care, and clinical services scores were consistently high (≥92%), consistent with the performance expectations set by the programme's competency framework at service delivery level. Safety — encompassing infection prevention and medical emergency management — was the lowest-scoring domain in both sub-periods (86–87%), corroborating the gap identified in the 2020 QTA (S3-04) and reaffirming that infection prevention and emergency preparedness remain high-priority areas within the programme's training and quality-improvement agenda. The proportion of sites meeting model-site criteria rose from 18% (2/11) in September–October 2021 to 39% (7/18) in the first half of 2022, consistent with a quality improvement trajectory across the network during the final phase of the study period.
