# Supplementary material for: Reconfiguration of clinical training delivery in a family planning and post-abortion care quality assurance programme in Sierra Leone, 2018–2022: a retrospective programme evaluation
Source: Glob Health Action. 2026 Nov 12;19(1):2761762. doi: 10.1080/16549716.2026.2761762 (PMC13761034; doi:10.1080/16549716.2026.2761762)
Supplement: Supplementary file 3 [file ZGHA_A_2761762_SM0834.docx]

Supplement S3: Public Sector Strengthening (PSS) baseline context and 2020 external QTA evidence

GHA 268442593 | Supplementary contextual evidence

# Purpose and distinctions

This supplement distinguishes (i) pre-service implementation readiness deficits, (ii) previously published programme-level outcome trends, and (iii) the nine-site independent external QTA snapshot. They are related sources, not interchangeable baselines or a facility-matched longitudinal cohort.

# A. Initial service-readiness context

According to programme management implementation experience, PSS sites were supported because personnel lacked prior training in the relevant FP/PAC services and essential service equipment was not yet available. Initial training was followed by provision of equipment and consumables, supervised practical service delivery, competency assessment and follow-up by MSSL/MoHS master trainers. These are historical implementation-context statements. The source material does not establish that each of the nine sites had a formally recorded L3 baseline classification; “untrained” must not be recoded as measured L3. A pre-service readiness assessment is different from a QTA based on observed clinical practice.

# B. Previously published PSS programme-level progression (2019–2020)

| Indicator | Earlier value | Later value |
| --- | --- | --- |
| Mean knowledge test score | 28% pre-training | 81% post-training |
| Level 1 competency proportion | 59% (Q3 2019) | 97% (Q4 2020) |
| Model-site status | 3% (2019) | 33% (2020) |
| Model-area status | 8% (2019) | 22% (2020) |
| PAC service volume | 442 (2019) | 1,950 (2020) |

Source: Onunkwor FI. From Training to Competency: An Operations Research Evaluation of Family Planning and Post-Abortion Care Capacity Building in Sierra Leone. Texila Int J Public Health. 2026;14(3). doi:10.21522/TIJPH.2013.14.03.Art012. Values are reported from the published abstract; do not infer matched individual or site-level change.

# C. External PSS QTA snapshot, March–April 2020

External assessment dates: 23 March–9 April 2020 (narrative report dated 14 April 2020). Eleven facilities were selected, nine were visited, and two could not be visited because of COVID-19 restrictions. The narrative report gives no prior site-specific QTA and does not document the randomisation method. The supplied nine-site score summary records three model sites. It reports clinical governance and safety scores, competency and supervision arrangements, and areas needing corrective action, notably infection-prevention practice and equipment constraints. The external QTA should not be merged numerically with the earlier published programme-level indicators.

# D. Strength of evidence and limits

- Pre-intervention service-readiness deficit: supported by programme account and separately published intervention description; not a measured pre-intervention QTA score.
- Published programme-level progression: peer-reviewed contextual longitudinal trends with different assessment units and denominators.
- External QTA: independent assessment following implementation at nine sites; not a paired longitudinal analysis or randomised treatment evaluation.
- The original nine-site report and score table contain facility names and assessor identifiers; a reviewer-facing copy should suppress facility identity and obtain necessary disclosure clearance.
- This supplement documents provenance and interpretation. It does not establish that any training component alone caused the observed scores or changes.
