# Supplementary material for: Reconfiguration of clinical training delivery in a family planning and post-abortion care quality assurance programme in Sierra Leone, 2018–2022: a retrospective programme evaluation
Source: Glob Health Action. 2026 Nov 12;19(1):2761762. doi: 10.1080/16549716.2026.2761762 (PMC13761034; doi:10.1080/16549716.2026.2761762)
Supplement: Supplementary file 4 [file ZGHA_A_2761762_SM0835.docx]

Supplementary evidence and denominator note

GHA 268442593 | Source-reconciliation note for author review and optional journal supplement

## Event classification

472 registered events: 380 clinical training; seven competency-assessed ToT; 85 non-competency workshops. Annual event counts 286, 52, 39, 76, 19 (2022 January–June).

## Channels

209 outreach, 172 centre and 91 PSS events; totals include all event types.

## Participant-episodes

3,817 across all events. In 387 competency-assessed activities: 2,571 participant-episodes; 2,559 with classified L1–L3 outcomes; 2,013 at L1 (78.7%). Twelve unclassified participant-episodes are excluded from the attainment denominator, not treated as L3 or failed assessments.

## Recorded client contacts

30,790 programme-recorded client-service encounters, not a verified direct-observation count; annual counts 10,862; 9,025; 6,032; 4,612; 259.

## Documentary alignment

18 quarterly supportive-supervision reports in the extraction matrix. No training event is documented in Q2 2018; routine CSS continued. The documentary review was first-author deductive extraction, not independently coded thematic analysis.

## Data availability

Raw trainer/staff-identifiable registers, underlying supervision narratives and client feedback are confidential. Controlled de-identified analytical disclosure remains subject to institutional authorisation and indirect identification safeguards.

This note is an aggregate summary, not a replacement for a fully audited, disclosable event-level supplementary dataset or for original approval documents.

## Annual competency numerator and denominator reconciliation

| Period | Level 1 numerator | L1+L2+L3 denominator | Attainment |
| --- | --- | --- | --- |
| 2018 | 491 | 799 | 61.5% |
| 2019 | 595 | 737 | 80.7% |
| 2020 | 561 | 593 | 94.6% |
| 2021 | 334 | 379 | 88.1% |
| Jan–Jun 2022 | 32 | 51 | 62.7% |
| Total | 2,013 | 2,559 | 78.7% |

All numerator and denominator figures are participant-episodes with recorded end-of-training classifications; annual figures are not adjusted for activity mix. This table supports the main manuscript Table 1 without claiming that these annual counts already appear there.

## PSS external quality assessment—interpretation

The external March–April 2020 nine-site QTA followed implementation of an integrated PSS service-readiness package. Lack of trained providers and essential equipment at initiation helps explain why directly comparable pre-intervention clinical QTA measurements were unavailable. Distinct published PSS programme-level 2019–2020 trends [21] must not be pooled with the nine-site external snapshot; see S3 evidence note.
